# Prolonged ictal and post‐ictal central apnea in an epileptic seizure

**DOI:** 10.1002/epd2.70247

**Published:** 2026-04-09

**Authors:** Wei Zhao, Alena Berkhamova, Rabeha Noor, Abdulrahman Alwaki, Elli Allen

**Affiliations:** ^1^ Texas Institute of Restorative Neurotechnologies (TIRN) University of Texas Health Science Center (UTHealth) Houston Texas USA; ^2^ Memorial Hermann Hospital – Texas Medical Center Houston Texas USA

**Keywords:** apnea, breathing belt, mesial temporal, SUDEP

A 36‐year‐old male, without conventional risk factors for epilepsy, developed seizure‐like episodes 2 years ago, characterized by loss of awareness and convulsions, for which he was referred to the epilepsy monitoring unit (EMU) for seizure characterization. In the EMU, multiple nocturnal focal left temporal seizures without bilateral tonic–clonic evolution (Figure 1, Figure S1) were captured, characterized by prolonged ictal and post‐ictal central apnea (ICA and PICA) lasting up to 52 and 25 s, respectively (Figure 1, Figures S2 and S3). Apnea was detected by the flattening of the thoracic and abdominal excursions monitored using respiratory inductance plethysmography (thoracic and abdominal belts).^1^ Brain MRI was normal, and serum autoimmune workup was negative. His seizures were well controlled on levetiracetam and lacosamide. Prolonged ICA and PICA have been identified as independent risk factors for sudden unexpected death in epilepsy^2^, ^3^ and ICA has been shown to be predictive of mesial temporal seizure onset.^1^

**FIGURE 1:**
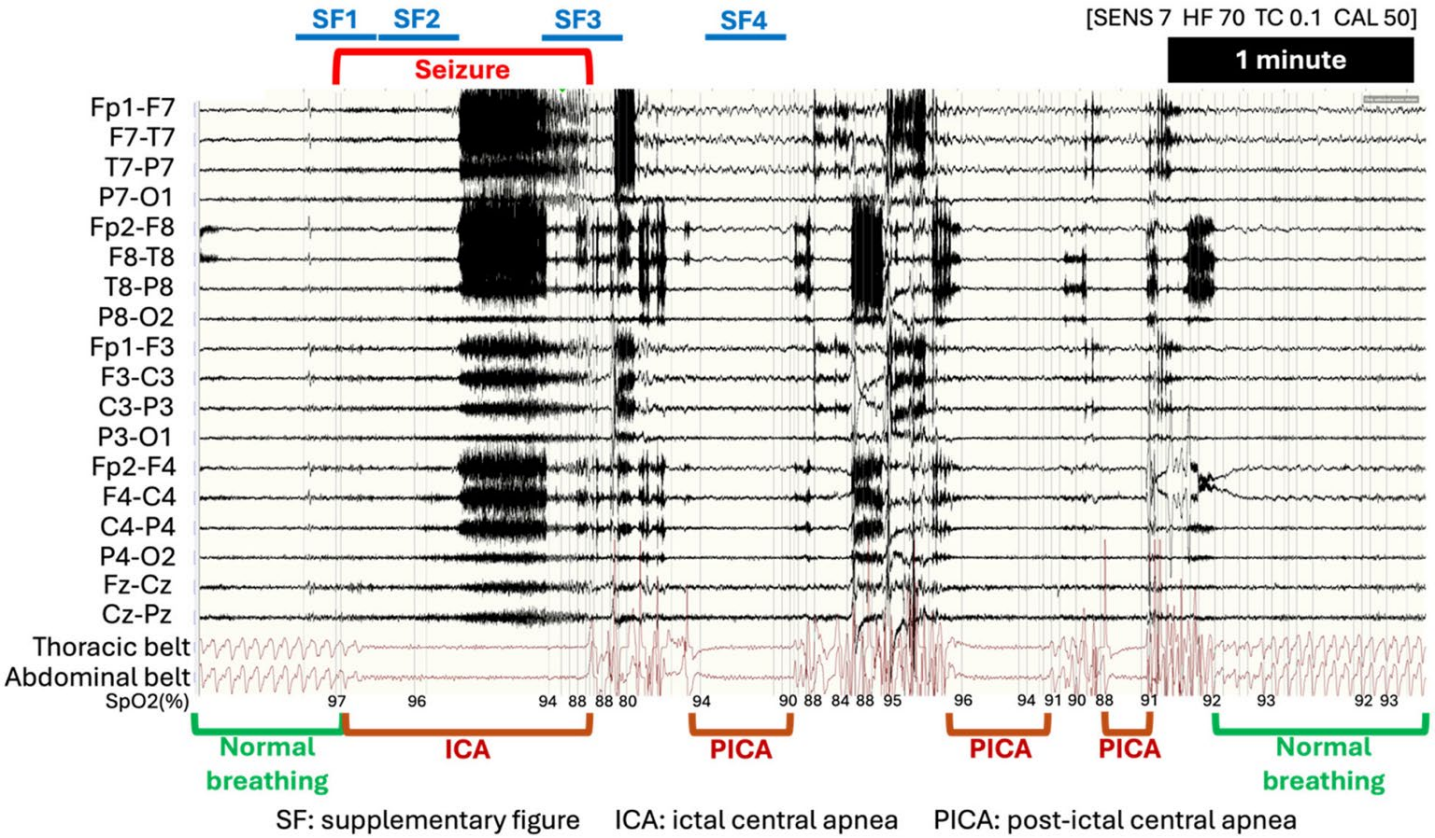
Five‐minute EEG plate with anterior‐posterior longitudinal bipolar (double‐banana) montage, thoracic‐ and abdominal‐belt tracings, and SpO2 tracing showing the seizure, ictal central apnea (ICA), post‐ictal central apnea (PICA), and the decrease in SpO2 lagging behind the onset of central apnea.

## FUNDING INFORMATION

The authors received no financial support for the research, authorship, and/or publication of this article.

## CONFLICT OF INTEREST STATEMENT

The authors declare no conflict of interest.

**Test yourself**

1. Ictal central apnea has localization value, and it has predictive value of seizure onset.
  - [A] Lateral temporal
  - [B] Mesial temporal
  - [C] Frontal
  - [D] Parietal
2. Which of the following has been verified in prospective studies as an independent risk factor for sudden unexpected death in epilepsy?
  - [A] Early‐onset (before 4 years old) of epilepsy
  - [B] Within the 15‐mile radius of a level‐IV epilepsy center
  - [C] Prolonged (>17 seconds) ictal central apnea and/or prolonged (>14 seconds) post‐ictal central apnea
  - [D] Daytime seizures
3. Which of the following risk factors has the largest effect on the risk of sudden unexpected death in epilepsy?
  - [A] Living alone
  - [B] Prolonged (>17 seconds) ictal central apnea
  - [C] Prolonged (>14 seconds) post‐ictal central apnea
  - [D] More than 3 generalized tonic‐clonic seizures in the previous year
*Answers may be found in the* *supporting information*.

## Supporting information


Figures S1–S4

Data S1

Data S2

## Data Availability

The data that support the findings of this study are available from the corresponding author upon reasonable request. The data are not publicly available due to privacy or ethical restrictions.
