# Supplementary material for: Prolonged ictal and post‐ictal central apnea in an epileptic seizure
Source: Epileptic Disord. 2026 Apr 9;28(4):1344–5. doi: 10.1002/epd2.70247 (PMC13499253; doi:10.1002/epd2.70247)
Supplement: Supplementary file 2 [file EPD2-28-1344-s002.docx]

**Supplementary figure 1**. Twenty-second EEG plate with anterior-posterior longitudinal bipolar (double-banana) montage and thoracic- and abdominal-belt tracings showing the seizure EEG onset in the left temporal region and the onset of apnea.

**Supplementary figure 2**. Twenty-second EEG plate with anterior-posterior longitudinal bipolar (double-banana) montage and thoracic- and abdominal-belt tracings showing the evolution of the ictal EEG pattern in the left temporal region, and the continuation of the ictal central apnea (ICA).

**Supplementary figure 3**. Twenty-second EEG plate with anterior-posterior longitudinal bipolar (double-banana) montage and thoracic- and abdominal-belt tracings showing the seizure EEG offset and ictal central apnea (ICA).

**Supplementary figure 4**. Twenty-second EEG plate with anterior-posterior longitudinal bipolar (double-banana) montage and thoracic- and abdominal-belt tracings showing the post-ictal slowing in the left temporal region and the post-ictal central apnea (PICA).
