# Supplementary material for: Prolonged ictal and post‐ictal central apnea in an epileptic seizure
Source: Epileptic Disord. 2026 Apr 9;28(4):1344–5. doi: 10.1002/epd2.70247 (PMC13499253; doi:10.1002/epd2.70247)
Supplement: Supplementary file 3 [file EPD2-28-1344-s001.docx]

Answers

1. [B]

2. [C]

3. [A]
